# The diagnostic utility of heparin-binding protein among patients with bacterial infections: a systematic review and meta-analysis

**DOI:** 10.1186/s12879-024-09004-w

**Published:** 2024-01-31

**Authors:** Amira Mohamed Taha, Khaled Abouelmagd, Mohamed Mosad Omar, Qasi Najah, Mohammed Ali, Mohammed Tarek Hasan, Sahar A. Allam, Roua Arian, Omar El Sayed Rageh, Mohamed Abd-ElGawad

**Affiliations:** 1 Faculty of Medicine, Fayoum University, Bank Street, Talat, Fayoum, Egypt; 2 Cardiology Department, Faculty of Medicine, Al-Azhar University, Cairo, Egypt; 3 Kasr Alainy School of Medicine, Cairo University, Cairo, Egypt; 4 Faculty of Medicine, University of EL-Mergib, Al Khums, Libya; 5 Faculty of Medicine, Al-Azhar University, Cairo, Egypt; 6 Faculty of Medicine, Tanta University, Tanta, Egypt; 7 Faculty of Medicine, University of Aleppo, Aleppo, Syria; 8 CME Office, Faculty of Medicine, University of Aleppo, Aleppo, Syria

**Keywords:** Heparin binding protein, Bacterial infections, Diagnostic accuracy, Biomarkers

## Abstract

**Background:**

Bacterial infections are considered a leading cause of hospitalization and death globally. There is still a need for a rapid and feasible biomarker for bacterial infections. Heparin-binding protein (HBP) was shown to be related to bacterial infections. The objective of the study is to investigate the diagnostic accuracy of HBP in bacterial infections.

**Methods:**

Articles were screened in PubMed, SCOPUS, Web of Science, and Cochrane to recognize eligible studies. We included studies investigating the diagnostic accuracy of HBP and reported the necessary data to construct 2 × 2 tables. A univariate analysis was conducted to determine the pooled sensitivity and specificity, and a bivariate diagnostic random-effects model was used to calculate the optimal cut-off point.

**Results:**

The analysis comprised sixteen studies in total. Plasma HBP showed a sensitivity of 0.90 (95% CI: [0.79, 0.96]) and a specificity of 0.87 (95% CI: [0.66, 0.96]) in diagnosing bacterial infections using blood samples. Pooling data from seven studies revealed that HBP in cerebrospinal fluid (CSF) has sensitivity and specificity of 96% (95% CI: [0.85, 0.99]), and 95% (95% CI: [0.89, 0.97]), respectively, for the diagnosis of bacterial meningitis. In urinary tract infections (UTI), urine-HBP was revealed to have a high diagnostic value in discriminating bacterial from non-bacterial UTI infection at a cut-off value of 32.868 ng/ml with sensitivity and specificity of 87%.

**Conclusion:**

HBP has shown a high diagnostic accuracy of bacterial infections, including UTI and meningitis. Further studies are needed to determine its prognostic value and whether it could guide antibiotic therapy.

**Supplementary Information:**

The online version contains supplementary material available at 10.1186/s12879-024-09004-w.

## Introduction

Bacterial infections are considered among the leading causes of hospitalization and death globally. Additionally, respiratory, urinary, and central nervous system bacterial infections are among the most common and serious bacterial infections in clinical settings [1].

Diagnosing bacterial infections is often challenging due to the similarities in the clinical picture of different infectious diseases [2]. Therefore, there is still a need for rapid, cheap, and feasible diagnostic techniques to tackle this issue, especially in low-income countries [3].

Moreover, using accurate diagnostic techniques is critical to avoid misdiagnosis, ineffective medications or antibiotics, and overprescribing antibiotics, which contribute to the development of antimicrobial resistance [2]. Previous observational studies have shown that up to 50% of prescribed antibiotics in clinics could be classified as unnecessary or inappropriate [4–7]. Therefore, developing rapid diagnostic tests and markers with sufficient accuracy would be necessary to improve clinical decision-making in antibiotic prescription and to limit the spread of antimicrobial resistance.

Various inflammatory markers, including procalcitonin (PCT), C-reactive protein (CRP), erythrocyte sedimentation rate (ESR), and interleukin-6 (IL-6), have been investigated for diagnosing bacterial infections [8]. Recently, Heparin-binding protein (HBP) was reported as a promising biomarker for the diagnosis of several infectious diseases.

HBP, also known as azurocidin, is a positively charged protein of 37 kDa that is stored in secretory and azurophilic granules and is rapidly mobilized upon stimulation of neutrophils in response to bacterial infection at early stages of inflammation. It plays a critical role in vascular leakage, extravasation of neutrophils, chemo-attraction, and activation of monocytes [9].

Recent findings showed that HBP is closely related to bacterial infections. Elevated levels of HBP in cerebrospinal fluid (CSF) were significantly associated with bacterial meningitis and proved to be a useful indicator for distinguishing between bacterial and non-bacterial forms of meningitis [10, 11].

Moreover, elevated urinary HBP was significantly associated with the presence of urinary tract infections (UTI) in adults and children [12, 13]. It was also reported to be of diagnostic value in respiratory tract infections (RTI) [14].

Thus, it is a promising rapid diagnostic marker for various bacterial infections for differentiating them from non-bacterial infections and aiding physicians in making appropriate treatment plans. However, the sample size in previous studies was limited, and most of the studies were single-center studies, so their findings may not be generalizable, and the diagnostic accuracy of HBP remains uncertain.

The objective of this study is to combine the existing evidence and examine the diagnostic value of HBP in different bacterial infections.

## Materials and method

### Literature search

The systematic review and meta-analysis were performed in accordance with the guidelines of the Preferred Reporting Items for Systematic Reviews and Meta-Analyses (PRISMA) guidelines [15]. We searched PubMed, Scopus, Web of Science, and Cochrane for relevant studies. The Medical Subject Headings (MeSH) terms and keyword search terms used were ("heparin-binding protein," OR "Heparin binding protein" OR "azurocidin”). All studies retrieved from these databases were assessed without limitations.

### Inclusion and exclusion criteria

Studies were included in the analysis if they evaluated the ability of HBP to diagnose bacterial vs. non-bacterial infections in adults accurately. The studies needed to provide enough data to construct a 2*2 table and to calculate true positives, true negatives, false positives, and false negatives.

Studies were excluded if they lacked the necessary data to construct a 2*2 table, did not directly compare bacterial and non-bacterial infections, were not written in English, or were reviews, correspondence, editorials, case reports, animal studies, or conference abstracts. The goal was to evaluate the diagnostic accuracy of high blood pressure in distinguishing bacterial from non-bacterial infections.

### Data extraction and quality assessment

The studies were evaluated by two authors independently who followed the inclusion and exclusion criteria. Data extraction and quality assessment were also done independently by two authors. They extracted information on study characteristics such as author, publication year, study design, country, and period. Patient characteristics such as eligibility criteria, patient source, type of sample, and time of collection, as well as clinical and demographic information of the patients, were also extracted, along with diagnostic criteria, outcomes, and accuracy parameters. Quality assessment was done using the Quality Assessment of Diagnostic Accuracy Studies (QUADAS-2) tool.

### Statistical analysis

Data were extracted and verified, then fed to R statistical software version 4.2.2 "Innocent and Trusting". A univariate analysis was done to determine the pooled sensitivity, specificity, and diagnostic odds ratio (DOR) using random effect models [16]. Additionally, a bivariate diagnostic random-effects meta-analysis was used to calculate the optimal cut-off point and the pooled area under the Summary Receiver Operating Characteristic (SROC) curve using the common random intercept method (CI) [17]. Mixed-effects models were conducted to examine the potential moderators explaining the heterogeneity in effect size between studies. The publication bias was also tested via a funnel plot Deek's test, after which the potential publication bias was adjusted using trim-and-fill methods, imputing studies that had been missed and then re-estimating the effect size after adjustment. Cochran's Q test was utilized to examine heterogeneity, and it was based on a chi-square distribution, and a *p*-value < 0.05 was considered statistically significant. The degree of heterogeneity was measured using the I2 index, and an I2 value of less than 40% indicated that the heterogeneity may not be significant. A value between 30% and 60% was considered moderate, between 50% and 90% indicated substantial heterogeneity, and an I2 value exceeding 75% was considered considerable heterogeneity [18].

## Results

### Summary of eligible studies

**Fig. 1:**
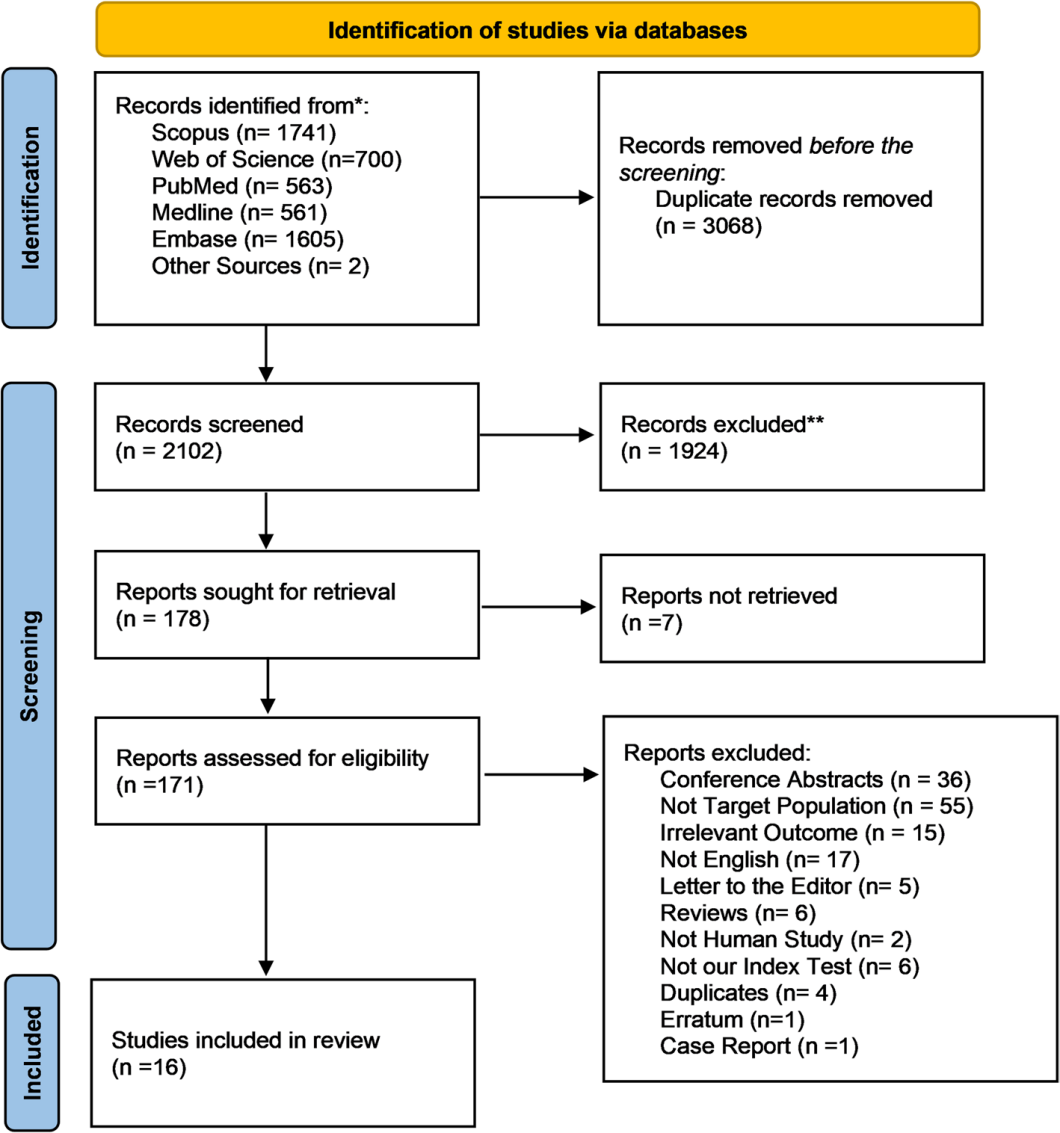
PRISMA Flow Diagram

After searching the literature, we identified 5170 studies, and two studies were retrieved by manual search. Among these studies, 3068 duplicates were removed, and 1931 were excluded by screening their titles and abstract, and the remaining 171 underwent further evaluation. After reading the full text of these articles, 155 studies were excluded. Thus, 16 studies met the inclusion criteria and were incorporated in the meta-analysis. The study selection process and causes of exclusion are shown in Fig. 1.

### Characteristics of included studies

Tables 1 and 2 list the characteristics of 16 included studies and patients. Fourteen studies included adult patients aged ≥ 18 years old (87.5%). Among the included studies, seven studies enrolled patients with central nervous system (CNS) infections, and four enrolled patients with urinary tract infections (UTI). In contrast, two studies enrolled patients with both bacterial and viral infections. The type of samples was either blood in eight studies [10, 11, 19–23], CSF in seven studies [10, 11, 21, 24–27], and urine in four studies [12–29]. HBP was assessed in different samples in all included studies.

**Table 1:** Characteristics of included studies. NA: Not available

**Study ID**	**Design**	**Country**	**Sample size**	**Inclusion criteria**	**Time of study conduction**	**Patient source or Study setting**	**Time of sample collection**	**Type of sample**	**Diagnostic criteria**	**Cut-off value**
**Cai 2021** [19]	Retrospective cohort study	China	141	Inclusion criteria: (1) age >18 years; (2) onset in the community; (3) new patchy infiltrates, lobar or segmental consolidation, ground-glass opacities or interstitial changes with or without pleural effusions; (4) new onset of cough or expectoration, or aggravation of existing symptoms of respiratory diseases.	From September 1, 2018, to May 31, 2019.	Nanjing First Hospital	Within 2 h after admission of patients	Blood	Diagnosis is according to the clinical manifestations, sputum culture and PCR results.	20.05
**Chalupa 2011** [20]	Prospective cohort study	Czech	81	Inclusion criteria: Adult patients (18–80 years old) presenting fever (axillary temperature C38 C) and a clinical diagnosis of infection (see below) were eligible for inclusion in this study if they were admitted to the standard wards of the Department of Infectious Diseases.	From April 2007 to September 2009.	Bulovka University Hospital	NA	Blood and Serum	Diagnosis of bacterial infection was made clinically based on the findings of focal infection.	NA
**Kandil 2018** [21]	Cross-sectional study	Egypt	90	Inclusion criteria: Patients with meningitis admitted to the Ministry of Health Specialized Hospital	End of 2016 to Early 2017	Fever Hospital in Alexandria	NA	Serum and CSF	Diagnosis was based on history, clinical criteria, and CSF examination criteria.	45.3 (Blood) 56.7 (CSF)
**Kjolvmark 2012** [13]	Prospective cohort study	Sweden	78	Inclusion criteria: elevated body temperature (≥37.5 °C) or symptoms suggesting UTI, such as abdominal or back pain in older children and irritability or feeding difficulties in younger children.	March to June 2009.	Hospital of Helsingborg	On admission	Urine	Diagnosis is based on patients with a final diagnosis of UTI based on a positive bacterial urine culture result [a single microorganism ≥105 colony-forming-units (CFU)/mL] and typical clinical symptoms of UTI.	32
**Kjolvmark 2014** [12]	Case control study	Sweden	345	Inclusion criteria: suspicion of UTI based on symptoms such as: dysuria, frequency, urgency, suprapubic pain, hematuria, and/or flank pain.	January to August 2012	Primary care and hospital ED, Hospital of Helsingborg	At the time of inclusion.	Urine and Blood samples	Diagnosis is based on clinical symptoms of UTI, bacterial species, and bacterial concentrations in the urine culture,	30
**Kjolvmark 2016** [28]	Prospective study	Sweden	87	NA	February to April 2013	Primary care and hospital ED, Hospital of Helsingborg	NA	Urine	Diagnosis is based on the results of the urine culture or presence of indwelling catheter.	30
**Kong 2022** [24]	Single-center observational study	China	281	Inclusion criteria: older than18 years and experienced neurosurgery that involved dura opening	August 2020 to June 2021	Beijing Tiantan Hospital	NA	CSF	Diagnosis is based on patients who stayed in the intensive care unit (ICU) for more than 48 h were defined as critically ill. Nosocomial meningitis or ventriculitis was confirmed when a patient met criteria 1 or criteria 2 of the definition.	23
**Lertdumrongluk 2015** [29]	Prospective study	Thailand	32	inclusion criteria: (1) urinary symptoms; (2) a previous diagnosis of UTI; (3) abnormalities in the urinary tract or obstructive uropathy; or (4) constitutional symptoms in children aged <3 years	January to September 2013	university-based tertiary care hospital	NA	Urine	Diagnosis is based on: Febrile patients with positive urine culture were classified as having APN. Positive urine culture was defined as single bacterial growth of ≥105 colony forming units (CFU)/mL from clean-voided mid-stream urine or ≥5×104 CFU/mL from catheterized urine.	34
**Linder 2011** [25]	Prospective and retrospective multicenter cohort study	Sweden	77	Inclusion criteria: patients with clinically suspected meningitis, who underwent a lumbar puncture	March 2006 to November 2009	Lund University Hospital, Lund, Sweden	NA	CSF	Diagnosis was based on classification of bacterial meningitis was based on the criteria of Durand.	20
**Ma 2022** [14]	Prospective case control study	China	87	Inclusion criteria: (I) inpatient with complete clinical data at the Department of Respiratory Medicine of our hospital; (II) be aged ≥18 years; (III) for the case group, meet the diagnostic criteria for respiratory tract bacterial infection	January 2019 to December 2019	Department of Respiratory Medicine of the East Hospital of Sichuan Provincial People’s Hospital	On hospital admission	Blood	Diagnosis was based on RTI on the definition and diagnostic criteria for RTI set out in the Guidelines for the Diagnosis and Treatment of Respiratory Diseases.	24.17
**Namiduru 2022** [11]	Prospective study	India	97	Inclusion criteria: Patients with meningitis admitted to University of Gaziantep Medicine Faculty Hospital.	January 2018 to June 2020	Department of Infectious Diseases and Clinical Microbi-ology Department of Gaziantep University Medical Faculty.	On inclusion	Blood and CSF	Diagnosis is based on bacterial meningitis is based on a course of clinical history and laboratory experiments. Clinical features were such as the acute onset of headache, fever, and signs of meningeal irritation. Laboratory diagnosis of acute bacterial meningitis (ABM) was made by CSF examination.	9.03 (Blood) 6.99 (CSF)
**Niu 2019** [22]	Randomized controlled trial	China	497	Inclusion criteria: suspected to be a bacterial or viral infection	October 2017 to February 2018	School of Laboratory Medicine and Life Sciences, Wenzhou Medical University	On inclusion	Blood	NA	3.83
**Obreja 2022** [10]	Prospective study	Romania	72	Inclusion criteria: over the age of 18 who presented signs and symptoms suggestive for meningitis and whose diagnoses were confirmed cytologically and biochemically by lumbar puncture	February 2018 to November 2020	Infectious Diseases Hospital	On admission	Blood and CSF	Diagnosis is based on identification of bacteria either directly by Gram stain smears and cultures from blood/CSF or indirectly by latex agglutination test of CSF confirmed the bacterial infections. Viral meningitis was defined as the presence of acute onset of meningitis symptoms.	2.47
**Ren 2021** [26]	Case control study	China	308	Inclusion criteria: for PM, were i) children with common symptoms of PM, including fever, irritability, vomiting, drowsiness, and impaired consciousness, and ii) positive bacterial cultures or smear test of the CSF. The inclusion criteria for VM were i) children	January 2018 to January 2020	Mianyang Central Hospital	NA	Blood and CSF	NA	54.7
**Yang 2022** [23]	Case control study	China	195	Inclusion criteria: (1) patients who met the diagnostic criteria for AURTI in children; (2) patients with no heart, liver, kidney or other important organ injuries; (3) patients without immune diseases and dermatomyositis; (4) patients aged 1–7.	September 2019 to January 2021	Department of Pediatrics of Fujian Maternity and Child Health Hospital	First day after admission. One day before discharge, the serum was tested again.	Blood	Diagnosis is based on patients who met the diagnostic criteria for AURTI in children after relevant inspections	NA
**Zhang 2019** [27]	Prospective study	China	94	Inclusion criteria: (1) patients with no heart, liver, kidney or other important organ injuries; (3) patients without immune diseases and dermatomyositis; (4) patients aged 1–7.	January to December 2016	Weifang People’s Hospital, Xuanwu Hospital Affiliated to Capital Medical University, and Qianfoshan Hospital of Shandong Province.	Within 72 hours.	CSF	Diagnosis is based on cerebrospinal fluid (CSF) WBC > 10 cells/μL with any of the following three items: (1) temperature (T) ≥ 38°C for more than 3 days; (2) meningeal irritation (+); (3) blood WBC > 10 × 109 cells/L; or CSF culture was positive.	NA

**Table 2:** Baseline of included studies, NA: Not available

Study ID	Study groups	Sample size	Age Mean (SD)	Gender (Male) n (total)	HBP at baseline Mean (SD)	Comorbidities n (%)
Kjolvmark 2012 [13]	Group 1	78	6.5 (5.04)	1 (10)	203 (187)	NA
	Group 2		7.7 (4.6)	1 (5)	113 (65)	NA
	Group 3		2 (4.25)	7 (30)	5 (29.5)	NA
	Group 4		7 (4.5)	17 (33)	4 (18.5)	NA
Kjolvmark 2014 [12]	Definite Cystitis PC	390	58 (54.81)	6 (105)	141.66 (149.62)	NA
	Definite Pyelonephritis PC		51.33 (49.62)	0 (12)	345.66 (322.96)	NA
	Probable Cystitis		54.33 (51.85)	1 (29)	98 (104.44)	NA
	Probable Pyelonephritis PC		71 (NA)	0 (1)	386 (NA)	NA
	No UTI PC		54.66 (51.11)	11 (47)	6.66 (6.66)	NA
	Controls		57.33 (17.77)	4 (25)	10 (11.11)	NA
	Definite Cystitis H		56 (62.29)	2 (13)	203.66 (308.97)	NA
	Definite Pyelonephritis H		59 (56.59)	26 (47)	236.33 (258.48)	NA
	Probable Cystitis H		51.33 (62.78)	3 (10)	92 (139.18)	NA
	Probable Pyelonephritis H		56.33 (55.3)	2 (5)	279.33 (681.81)	NA
	No UTI H		59 (55.7)	51 (96)	7.66 (6.77)	NA
Kjolvmark 2016 [28]	Asymptomatic bacteriuria	163	87.66 (5.39)	4 (38)	102.33 (151.77)	Urogenital disease 5 (13) , Malignancy 9 (24) , Diabetes mellitus 4 (11), Chronic obstructive disease 4 (11)
	Urinary tract infection		87 (7.63)	20 (49)	257.33 (3.11)	
	Indwelling catheter		87.33 (8.044)	15 (18)	412 (320.98)	Urogenital disease 9 (50), Malignancy 6 (33), Diabetes mellitus 6 (33), Chronic obstructive disease 1 (6)
	Negative culture		87 (8.36)	15 (57)	11.33 (17.49)	Urogenital disease 5 (9), Malignancy 7 (12), Diabetes mellitus 8 (14), Chronic obstructive disease 3 (5)
Kong 2022 [24]	Infected group	323	49 (44.97)	64 (131)	Culture-positive group 162.66 Culture-negative group 132.66 (culture-positive group 72.66 culture negative group 80.53)	Cardiovascular disease: 33 (25), Respiratory disease 6 (5), Endocrine disease 12 (9), Central nervous system disease 29 (22), Digestive system disease 8 (6), No medical history 43 (33)
	Control group		49 (41.9)	62 (151)	NA	Cardiovascular disease: 39 (26), Respiratory disease 3 (2), Endocrine disease 10 (7%), Central nervous system disease 34 (23), Digestive system disease 12 (8), No medical history 48 (32)
Linder 2011 [25]	Bacterial Meningitis	174	51 (16)	20 (41)	415.33 (649.93)	21 (41.1)
	Viral Encephalitis		55 (13)	10 (19)	16.33 (30.43)	4 (19)
	Viral Meningitis		43 (17)	6 (10)	15.76 (31.73)	1 (10)
	Neuroborreliosis		53 (16)	3 (7)	5.6 (6.2)	2 (6.7)
Namiduru 2022 [11]	Bacterial meningitis	97	40.19 (2.73)	18 (37)	Serum HBP 14.98 – CSF HBP 7.81 (Serum HBP 1.1 - CSF HBP 0.2)	NA
	Tuberculosis meningitis		42.6 (3.31)	17 (30)	Serum HBP 6.89 - CSF HBP 6,11 (Serum HBP 0.4 - CSF HBP 0.3)	NA
	Viral meningitis		45.4 (2.56)	19 (30)	Serum HBP 6.02 - CSF HBP 5.75 (Serum HBP 0.4 - CSF HBP 0.1)	NA
Obreja 2022 [10]	Bacterial Meningitis	81	52.72 (20.03)	29 (47)	HBP in CSF 66.00 HBP in blood: 4.86 (HBP in CSF 134.50 HBP in blood: 6.71)	Alcoholism 21 (44.7) Smoking 7 (14.9)
	Viral Meningitis		57.2 (16.6)	22 (34)	HBP in CSF 2.38 HBP in blood: 18.88 (HBP in CSF 5.63 HBP in blood: 58.13)	Alcoholism 12 (35.3) Smoking 9 (26.5)
Zhang 2019 [27]	BII	134	35.9 (16.8)	23 (40)	88.1 (38.2)	NA
	NBII		36.9 (17.6)	31 (54)	30.1 (14.6)	NA
	Control		36.2 (17.2)	19 (40)	23.56 (11.2)	NA
Kandil 2018 [21]	Bacterial group	90	24.7 (14.7)	19 (30)	192.2 (56.6)	NA
	Viral group		24.7 (14.8)	18 (30)	3.7 (1.9)	NA
	Control group		24.9 (14.3)	12 (30)	0.84 (0.3)	NA
Yang 2022 [23]	Research group (bacterial & viral infection groups)	195	≥5 years: 62(47.69) <5 years: 68 (52.31)	81 (130)	Bacterial group: 31.58 (5.03). Viral: 25.21 (2.73)	NA
	Control group		≥5 years: 32 (49.23) <5 years: 33 (50.77)	37 (65)	3.23 (0.82)	NA
Niu 2019 [22]	Bacterial group	497	NA	NA	62.1 (57.2)	NA
	Viral group		NA	NA	9 (3.5)	NA
	Sepsis		NA	NA	92.8 (37.6)	NA
	Tumor		NA	NA	13.9 (10.6)	NA
	Cardiovascular Diseases		NA	NA	27 (35.6)	NA
Ren 2021 [26]	Purulent Meningitis	308	3.6 (0.4)	60 (118)	NA	NA
	Viral Meningitis		3.7 (0.5)	63 (110)	NA	NA
	Control group		3.2 (0.6)	41 (80)	NA	NA
Chalupa 2011 [20]	Bacterial Infections group	81	46.8 (18.2)	27 (54)	51 (31.9891)	NA
	Viral infections group		42.8 (15.2)	18 (27)	21 (7.0441)	NA
Lertdumrongluk 2015 [29]	APN	32	1.6 (1.075)	12 (17)	NA	NA
	Control group		4.55 (2.96)	9 (15)	NA	NA
Cai 2021 [19]	Bacterial group	102	69.93 (17.28)	71(108)	53.653 (33.79)	NA
	Fungal group		67.76 (17.78)	12 (21)	62.47 (93.409)	NA
	Viral group		64.79 (21.1)	19 (33)	11.727 (6.285)	NA

### Assessment of risk of bias

**Fig. 2:**
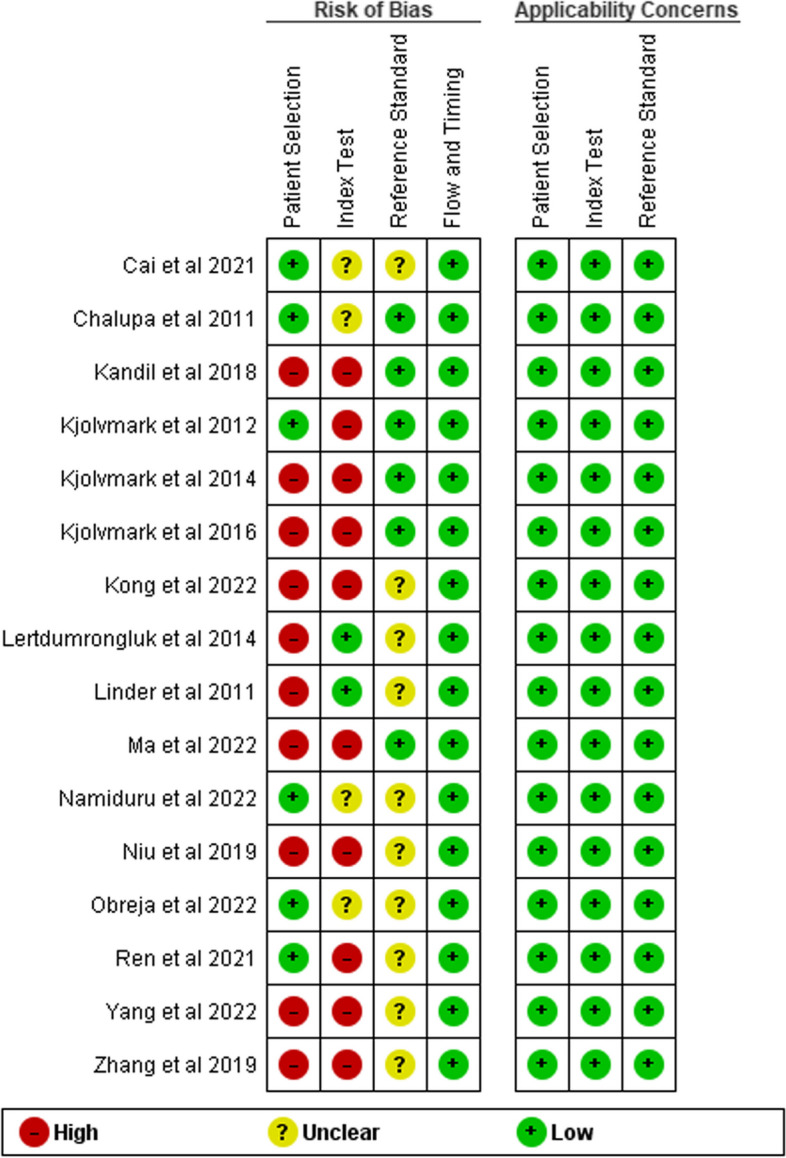
Quality Assessment of Included Eligible Studies Using QUADAS-2; Risk of Bias Summary

Figure 2 displays the risk of bias assessment details. In the patient selection domain, ten studies (62.5%) had high-risk patient selection bias, primarily due to the use of a case-control study design [12, 21–25, 27–29], or inappropriate patient selection [11, 12, 21–25, 27–29]. In the index test domain, ten studies (62.5%) had a high risk of bias as they lacked a pre-specified cut-off threshold or interpretation bias [12, 21–24, 26–28]. For the reference standard domain, ten studies (62.5%) had unclear risk of bias due to interpretation bias or lack of knowledge of index test results [10, 11, 19, 22–27, 29]. The risk of bias for the flow and timing domain was low in all studies. None of the studies had any concerns for applicability in any domain, whether high or unclear.

### Meta-analysis of the diagnostic accuracy of hbp in the diagnosis of bacterial infections

#### Plasma HBP levels and the diagnosis of bacterial infections

##### Univariate analysis and meta-regression

**Fig. 3:**
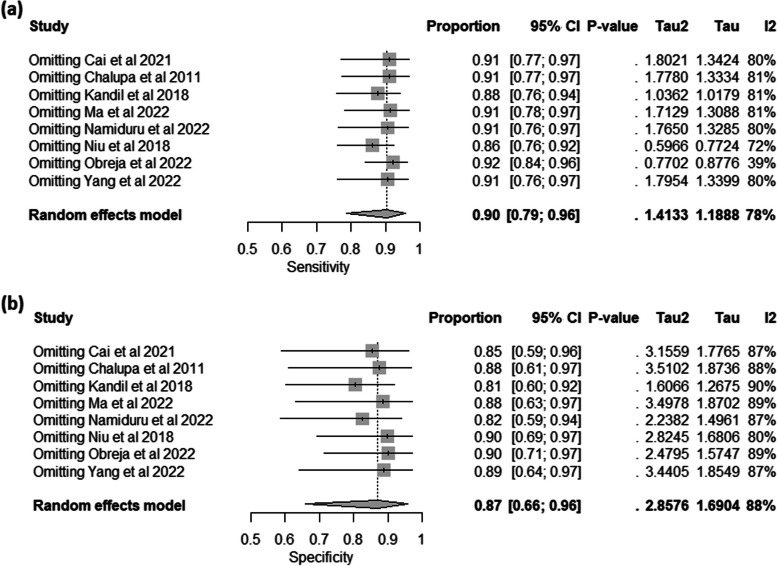
The Univariate Analysis for Plasma HBP in Diagnosing Bacterial Infections; (**a**) Forest Plot of Pooled Sensitivity After the Leave-One-Out test; (**b**) Forest Plot of Pooled Specificity After the Leave-One-Out test

The analysis involved eight individual studies investigating the diagnostic accuracy of HBP in plasma in cases of bacterial infection. The random-effects meta-analysis model showed that the pooled sensitivity was 0.90 with 95%CI: [0.79, 0.96], the between-study heterogeneity was considerable (I^2 =78%), heterogeneity variance (tau^2 = 1.4133), and there was a significant test for heterogeneity (*p*< 0.01) (Figure S1-a). Leave-one-out test showed that the heterogeneity would be resolved by omitting Obreja et al. 2022 study (0.92, 95% CI [0.84, 0.96], I^2 = 39%) (Fig. 3a). The pooled specificity was 0.87 with 95%CI: [0.66, 0.96], the between-study heterogeneity was considerable (I^2 =88%), a heterogeneity variance (tau^2 = 2.8576), and there was also a significant test for heterogeneity (*p*< 0.01) (Figure S1-b). However, the heterogeneity was not resolved by conducting the leave-one-out test (Fig. 3b).

The pooled DOR was 48.04 with 95% CI: [9.50, 242.85], the between-study heterogeneity was (I^2 = 89%), a heterogeneity variance (tau^2 = 4.6388), and the heterogeneity test was significant (p< 0.01) (Figure S2-a). The heterogeneity was not resolved by the leave-one-out test (Figure S2-b).

The age, gender, HBP at baseline, the used cut-off values, and the publication year have been considered non-statistically significant moderators for the between-studies heterogeneity in effect size (Table S1, Figure S3).

##### Bivariate diagnostic random effects

**Fig. 4:**
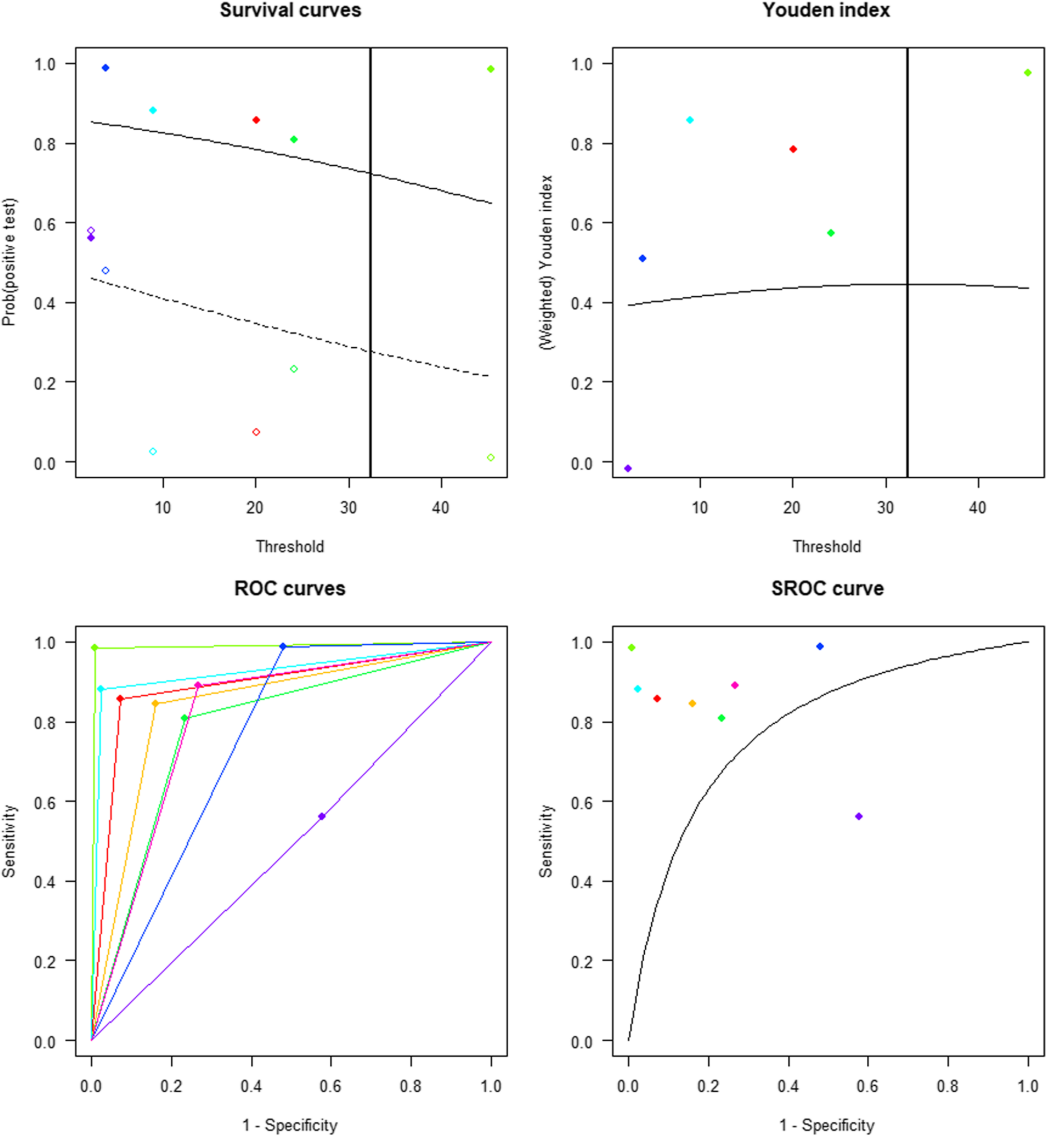
The Optimal Cut-off Value of Plasma HBP Used for Early Diagnosis of Bacterial Infections; (**a**) Kaplan–Meier Curves (**b**) Youden’s index derived Optimal Cut-off Value of Plasma HBP of 32.381 ng/mL; (**c**) ROC Curve; (**d**) The Summary Receiver Operator Characteristic (SROC) Curve for Plasma HBP

At the cut-off point of 32.381 ng/ml, the sensitivity and specificity were 0.7231, 95% CI [0.3166, 0.9364] and 0.7231, 95% CI [0.2794, 0.9462], respectively. The pooled AUC was 0.7853 with 95% CI [0.2642, 0.9780] (Fig. 4).

#### CSF HBP levels and the diagnosis of CNS infections

##### Univariate analysis and meta-regression

**Fig. 5:**
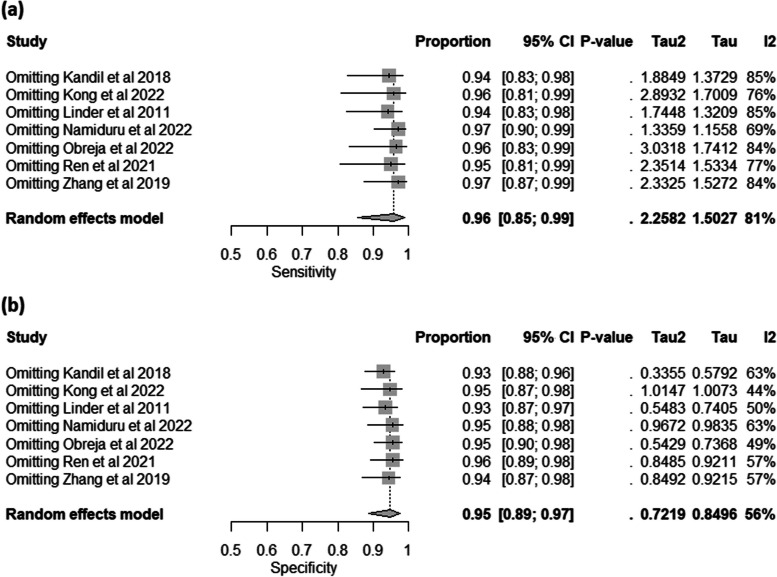
The Univariate Analysis for CSF HBP in Diagnosing CNS Infections; (**a**) Forest Plot of Pooled Sensitivity After the Leave-One-Out test; (**b**) Forest Plot of Pooled Specificity After the Leave-One-Out test

The analysis included seven studies examining the diagnostic utility of HBP in the CSF of patients with CNS infections, mostly meningitis. The random-effects meta-analysis model revealed a pooled sensitivity of 0.96 with 95% CI: [0.85, 0.99], the between-study heterogeneity was considerable (I^2= 81%), a heterogeneity variance (tau^2 = 2.2582), and a significant test for heterogeneity (*p* < 0.01) (Figure S4-a). The heterogeneity was not resolved by the leave-one-out test (Fig. 5a). The pooled specificity was 0.95 with 95% CI: [0.89, 0.97], the between-study heterogeneity was moderate (I^2= 56%), the heterogeneity variance was 0.7219, and the heterogeneity test was significant (*p* = 0.04) (Figure S4-b). Leave one out test showed that the heterogeneity resolved after omitting the Kong et al. 2022 study (0.95, 95% CI [0.87, 0.98], I^2 = 44%) (Fig. 5b).

The pooled DOR was 234.53 with 95% CI: [56.04, 981.45], the between-study heterogeneity was considerable (I^2= 80%), the heterogeneity variance was 2.7402, and there was a significant test for heterogeneity (p < 0.01) (Figure S5-b). The heterogeneity was not resolved by the leave-one-out test (Figure S5-b).

The meta-regression analysis has revealed that the HBP at baseline can be considered as a statistically significant moderator for the between-studies heterogeneity in effect size, and there was 67.21% residual heterogeneity after including the HBP at baseline as a covariate (*p*-value= 0. 0268) (Table S2, Figure S6).

#### Urinary HBP and the Diagnosis of UTI

##### Univariate analysis and meta-regression

**Fig. 6:**
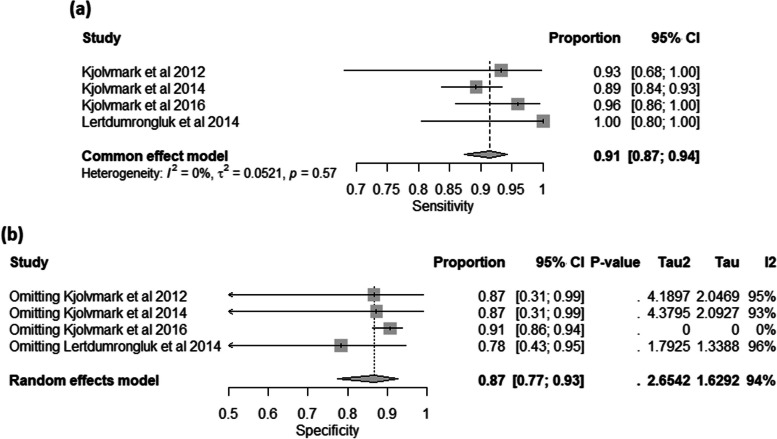
The Univariate Analysis for Urinary HBP in Diagnosing Urinary Tract Infections; (**a**) Forest Plot of Pooled Sensitivity; (**b**) Forest Plot of Pooled Specificity After the Leave-One-Out test

The analysis involved four individual studies investigating the diagnostic accuracy of urine-HBP in cases of bacterial infection. The fixed-effects meta-analysis model showed that the pooled sensitivity was 0.91 with 95% CI: [0.87, 0.94], the between-study heterogeneity was not significant (I^2 =0%), heterogeneity variance (tau^2 = 0.0521), and there was an insignificant test for heterogeneity (*p* = 0.57) (Fig. 6a). The pooled specificity was 0.87 with 95%CI: [0.77, 0.93], the between-study heterogeneity was considerable (I^2 =94%), a heterogeneity variance (tau^2 = 2.6542), and there was also a significant test for heterogeneity (*p*< 0.01) (Figure S7). The heterogeneity was resolved by omitting the Kjolvmark et al. 2016 study (0.91, 95% CI [0.86, 0.94], I^2 = 0%) (Fig. 6b).

The pooled DOR was 63.35 with 95% CI: [17.05, 235.42], the between-study heterogeneity was moderate (I^2 = 56%), a heterogeneity variance (tau^2 = 0.9379), and the heterogeneity test was significant (*p* = 0.08) (Figure S8-b). The heterogeneity was resolved by leaving out Kjolvmark et al. 2016 study (83.57, 95% CI [43.60, 160.16], I^2 = 0%) (Figure S8-b).

2. Bivariate Diagnostic Random Effects

**Fig. 7:**
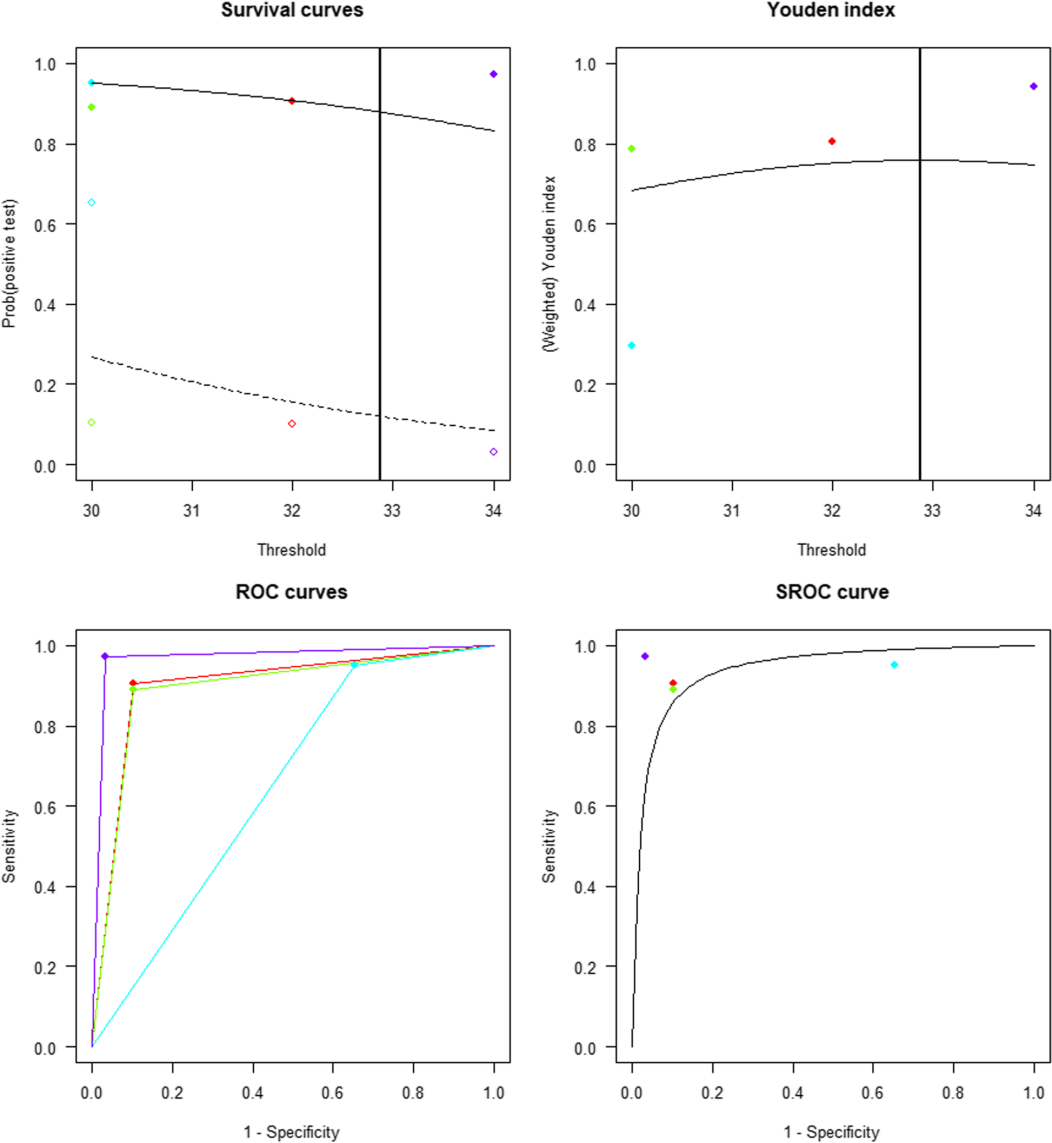
The Optimal Cut-off Value of Urinary HBP Used for Early Diagnosis of Urinary Tract Infections; (**a**) Kaplan–Meier Curves (**b**) Youden’s index derived Optimal Cut-off Value of HBP of 32.868 ng/mL; (**c**) ROC Curve; (**d**) SROC Curve for Urinary HBP

At the cut-off value of 32.868 ng/ml, the sensitivity and specificity were 0.8795, 95% CI [0.3731, 0.9889] and 0.8795, 95% CI [0.3969, 0.9878], respectively. The pooled AUC was 0.9416 with 95% CI [0.3156, 0.9972] (Fig. 7).

## Discussion

Bacterial infection is recognized as a triggering cause of various complications, including hepatic encephalopathy, liver and renal failure, coagulation disorders, and death. Therefore, early detection of bacterial infections is critical but difficult due to the similarities in the clinical presentation of different infectious diseases. Also, the bacterial culture, which is the gold standard diagnostic test for bacterial infections, has poor sensitivity and delayed results.

Therefore, a rapid and more accurate laboratory biomarker is required. HBP could be considered an easy and rapid laboratory test with potential diagnostic value in bacterial infections. The results of the following meta-analysis, including 16 studies, indicated that HBP is an effective biomarker for the diagnosis of different bacterial infections, including UTI and CNS infections while discriminating them from non-bacterial infections.

CSF analysis is considered the gold standard for the confirmation of a suspected case of bacterial meningitis [30]. Pooling data from seven studies resulted in an HBP sensitivity and specificity of 96% and 95% for the diagnosis of bacterial meningitis. Additionally, the diagnostic accuracy of elevated CSF HBP appeared to be superior to blood HBP.

Several of the studies included in the analysis evaluated the diagnostic effectiveness of HBP in conjunction with other biomarkers or compared it to them in the diagnosis of CNS infections. Kong et al. showed that CSF HBP concentrations were superior to CSF PCT or lactate concentrations in the identification of nosocomial meningitis or ventriculitis, suggesting its utility in the early identification of patients with bacterial infections. The sensitivity and negative predictive value of HBP were higher than lactate. At the same time, the specificity was lower than it, indicating that CSF HBP is more valuable for confirmation of the presence of infection with a low risk of missed diagnosis [24].

Whereas lactate would be more suggestive of an active infection, with a low probability of misdiagnosis. On the contrary, CSF PCT revealed poor sensitivity among included patients, and PCT concentrations were normal in some patients despite being diagnosed with meningitis or ventriculitis, suggesting that CSF PCT values have little clinical utility and can be used for the exclusion of nosocomial infections. So, only the early and simultaneous measurement of CSF HBP and lactate biomarkers was suggested to be more clinically useful in cases suspected of nosocomial meningitis or ventriculitis [24].

The lack of diagnostic utility of CSF PCT in the diagnosis of bacterial intracranial infection was also confirmed by Zhang et al. The author revealed that the AUC of HBP was greater than that of PCT alone or in combination with HBP [27].

CSF HBP was found to be a superior diagnostic tool for bacterial meningitis than other biomarkers, such as neutrophil gelatinase-associated lipocalin (NGAL) and S100 calcium-binding protein B (S100B). CSF HBP demonstrated an exceptional ability to differentiate between bacterial and viral meningitis [10].

Moreover, serum and CSF HBP levels were higher in children with purulent meningitis than those with viral meningitis compared to other infection biomarkers, including PCT, CRP, and tumor necrosis factor (TNF)-α. Additionally, HBP showed the highest diagnostic value among those four biomarkers [26].

In UTI, we found that the urine-HBP is of high diagnostic value in discriminating bacterial from non-bacterial UTI infection at a cut-off value of 32.868 ng/ml with sensitivity and specificity and pooled AUC of 87%, 87%, and 94%, respectively.

Urine-HBP showed a higher specificity than white blood cell count and Il-6 in the diagnosis of UTI and greater sensitivity than nitrite in children [13]. However, it showed a low discriminatory value between the elderly with UTI and those with asymptomatic bacteriuria compared to IL-6 despite having a higher negative predictive value (93.5% vs. 74-76% for urine-HBP and urine IL-6, respectively).

This could be explained by the elevated urine HBP in both patients with asymptomatic bacteriuria due to the inflammatory response and excess neutrophil lysis and those with UTI due to the pro-inflammatory response and excess HBP release. At the same time, IL-6 is lower in patients with asymptomatic bacteriuria due to the absence of a pro-inflammatory IL-6 response in contrast to those with UTI. However, urine HBP could still be considered a potential biomarker for ruling out UTI [28].

## Strengths and limitations of the study

Regarding the strengths, we conducted bivariate models which are significantly associated with the low influence of threshold effects [31]. Additionally, the diagnostic accuracy of HBP in various bacterial infections was reviewed for the first time through the current systematic review and meta-analysis.

One of the limitations of our study was that we restricted our search to studies published in English, which may limit the applicability of our results. Additionally, there was notable heterogeneity among the included studies that we tried to address its source by leave-one-out test. The patient population, testing interval time, and cut-off value used may have contributed to the detected heterogeneity. Some of the studies have reported their results insufficiently, thus impacting data extraction and quality assessment. Additionally, the included studies were characterized by small sample sizes and different study populations, which may impact the immune response to infections. Three out of four UTI patient studies were authored by the same researcher, raising concerns about potential duplication and over-representation. However, we conducted a thorough investigation to detect any such duplications, which were not found. This was supported by the fact that these studies were carried out in different years and with different inclusion criteria and study design. Furthermore, we performed a thorough sensitivity analysis to ensure the robustness of our findings.

## Conclusion

In summary, the available data support the diagnostic utility of HBP levels in the diagnosis of bacterial infections. Our analysis supports the high diagnostic accuracy of HBP in the blood, urine, or CSF in diagnosing UTI and CNS infections. However, the diagnostic value of HBP, along with other biomarkers such as PCT, CRP, or IL-6, as well as the specific time for the test, would require further investigations. Additionally, more studies are needed to determine if HBP levels are correlated with the prognosis of bacterial infections and whether they can be used safely and effectively to guide antibiotic therapy.

### Supplementary Information

**Additional file 1:** **Figure S1.** The Univariate Analysis for Plasma HBP in Diagnosing Bacterial Infections Before Leave-one-out Test; (a) Forest Plot of Pooled Sensitivity; (b) Forest Plot of Pooled Specificity.**Figure S2.** (a) Forest Plot of the Diagnostic Odds Ratio (DOR) of Plasma HBP for the Diagnosis of Bacterial Infections; (b) Forest Plot of the DOR of Plasma HBP for the Diagnosis of Bacterial Infections After the Leave-one-out Test. **Table S1.** Results of Meta-regression Analysis of Studies Investigating Plasma HBP. **Figure S3.** Deek's Funnel Plot Showing the Effect of HBP Cut-off values on the Effect Size in Studies Investigating Plasma HBP. **Figure S4.** The Univariate Analysis for CSF HBP in Diagnosing CNS Infections Before Leave-one-out Test; (a) Forest Plot of Pooled Sensitivity; (b) Forest Plot of Pooled Specificity. **Figure S5.** (a) Forest Plot of the diagnostic Odds Ratio (DOR) of CSF HBP for the Diagnosis of CNS Infections; (b) Forest Plot of the DOR of CSF HBP for the Diagnosis of CNS Infections After the Leave-one-out Test. **Table S2.** Results of Meta-regression Analysis of Studies Investigating CSF HBP. **Figure S6.** Deek's Funnel Plot Showing the Effect of HBP at Baseline on the Effect Size in Studies Investigating CSF HBP. **Figure S7.** Forest Plot of Pooled Specificty of Urinary HBP in Diagnosing Urinary Tract Infections Before Leave-one-out Test.** Figure S8.** (a) Forest Plot of the diagnostic Odds Ratio (DOR) of Urinary HBP for the Diagnosis of Urinary Tract Infections; (b) Forest Plot of DOR of Urinary HBP for the Diagnosis of Urinary Tract Infections After the Leave-one-out Test.

## Data Availability

All data generated or analyzed during this study are included in this published article or the data repositories listed in References.
