# Supplementary material for: The diagnostic utility of heparin-binding protein among patients with bacterial infections: a systematic review and meta-analysis
Source: BMC Infect Dis. 2024 Jan 31;24:150. doi: 10.1186/s12879-024-09004-w (PMC10829335; doi:10.1186/s12879-024-09004-w)
Supplement: Supplementary file 1 — Additional file 1: Figure S1. The Univariate Analysis for Plasma HBP in Diagnosing Bacterial Infections Before Leave-one-out Test; (a) Forest Plot of Pooled Sensitivity; (b) Forest Plot of Pooled Specificity.Figure S2. (a) Forest Plot of the Diagnostic Odds Ratio (DOR) of Plasma HBP for the Diagnosis of Bacterial Infections; (b) Forest Plot of the DOR of Plasma HBP for the Diagnosis of Bacterial Infections After the Leave-one-out Test. Table S1. Results of Meta-regression Analysis of Studies Investigating Plasma HBP. Figure S3. Deek's Funnel Plot Showing the Effect of HBP Cut-off values on the Effect Size in Studies Investigating Plasma HBP. Figure S4. The Univariate Analysis for CSF HBP in Diagnosing CNS Infections Before Leave-one-out Test; (a) Forest Plot of Pooled Sensitivity; (b) Forest Plot of Pooled Specificity. Figure S5. (a) Forest Plot of the diagnostic Odds Ratio (DOR) of CSF HBP for the Diagnosis of CNS Infections; (b) Forest Plot of the DOR of CSF HBP for the Diagnosis of CNS Infections After the Leave-one-out Test. Table S2. Results of Meta-regression Analysis of Studies Investigating CSF HBP. Figure S6. Deek's Funnel Plot Showing the Effect of HBP at Baseline on the Effect Size in Studies Investigating CSF HBP. Figure S7. Forest Plot of Pooled Specificty of Urinary HBP in Diagnosing Urinary Tract Infections Before Leave-one-out Test. Figure S8. (a) Forest Plot of the diagnostic Odds Ratio (DOR) of Urinary HBP for the Diagnosis of Urinary Tract Infections; (b) Forest Plot of DOR of Urinary HBP for the Diagnosis of Urinary Tract Infections After the Leave-one-out Test. [file 12879_2024_9004_MOESM1_ESM.docx]

**Supplementary Figures and Tables**


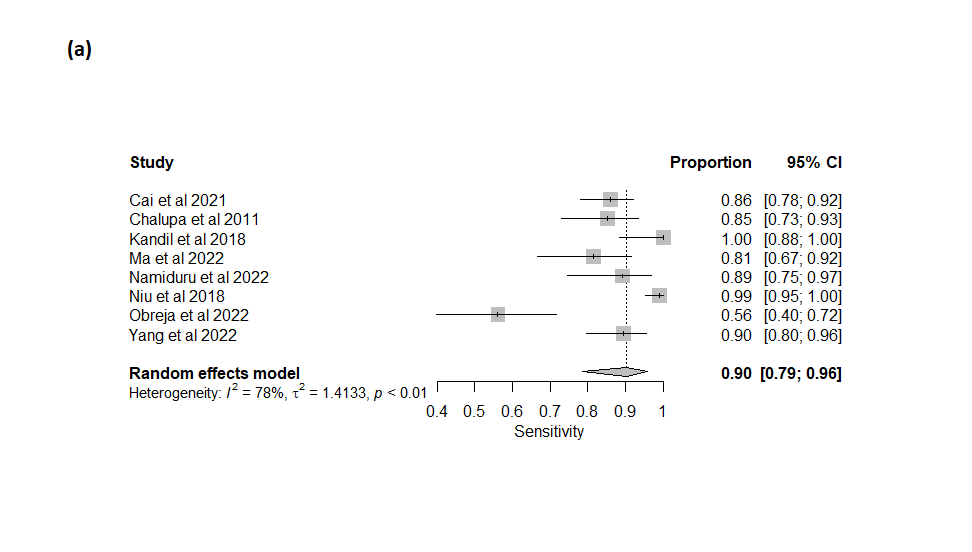

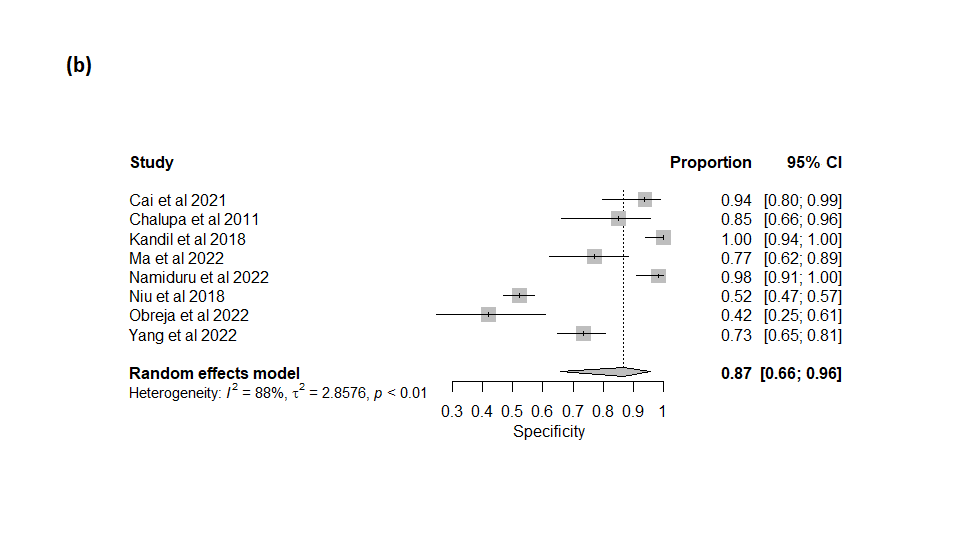


Figure S1. The Univariate Analysis for Plasma HBP in Diagnosing Bacterial Infections Before Leave-one-out Test; (a) Forest Plot of Pooled Sensitivity; (b) Forest Plot of Pooled Specificity.


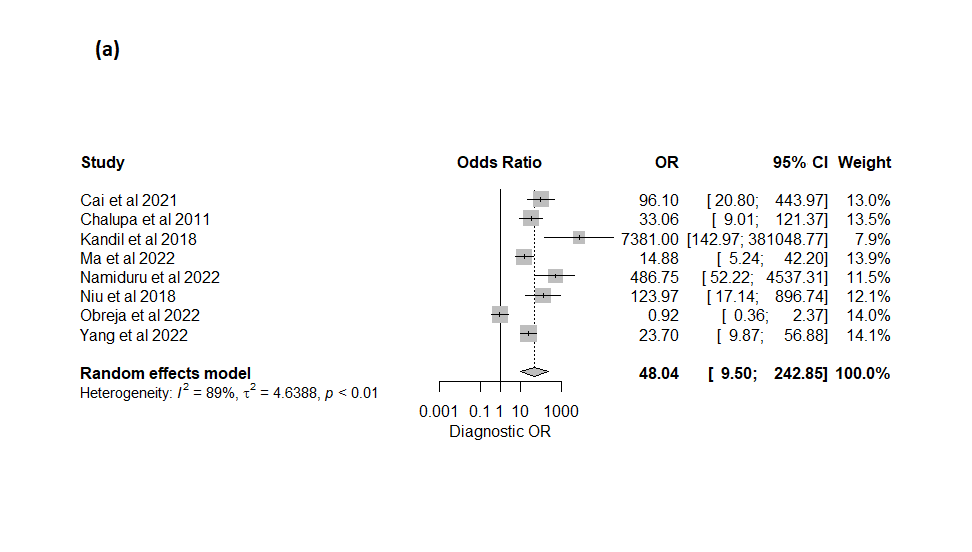

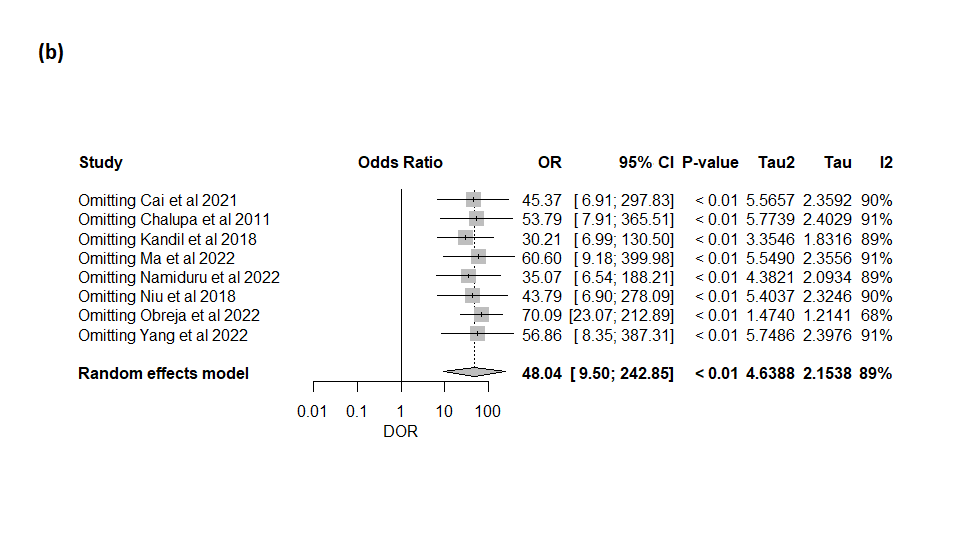


Figure S2. (a) Forest Plot of the Diagnostic Odds Ratio (DOR) of Plasma HBP for the Diagnosis of Bacterial Infections; (b) Forest Plot of the DOR of Plasma HBP for the Diagnosis of Bacterial Infections After the Leave-one-out Test.

Table S1. Results of Meta-regression Analysis of Studies Investigating Plasma HBP

| Covariate | I^2^ after (%) | Coefficient | SE of coefficient | p value |
| --- | --- | --- | --- | --- |
| **Year** | 92.13 | -0.1155 | 0.2407 | 0.6313 |
| **Age** | 92.97 | -0.1133 | 0.0857 | 0.1860 |
| **Male** | 93.64 | -0.0104 | 0.0471 | 0.8261 |
| **Female** | 92.65 | 0.0354 | 0.0705 | 0.6162 |
| **HBP at baseline** | 91.72 | 0.0833 | 0.0547 | 0.1277 |
| **Cutoff** | 89.25 | 0.1178 | 0.0802 | 0.1421 |


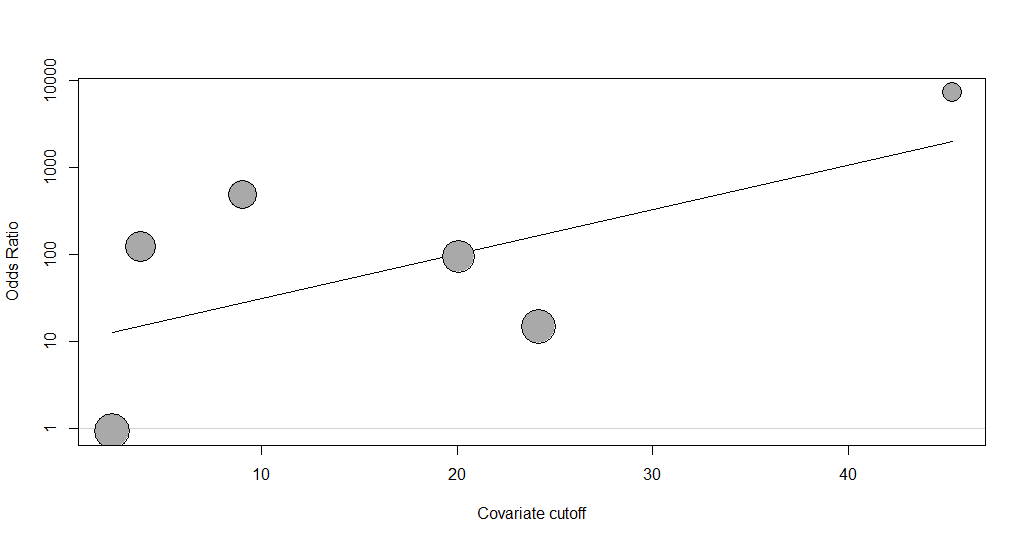


Figure S3. Deek's Funnel Plot Showing the Effect of HBP Cut-off values on the Effect Size in Studies Investigating Plasma HBP


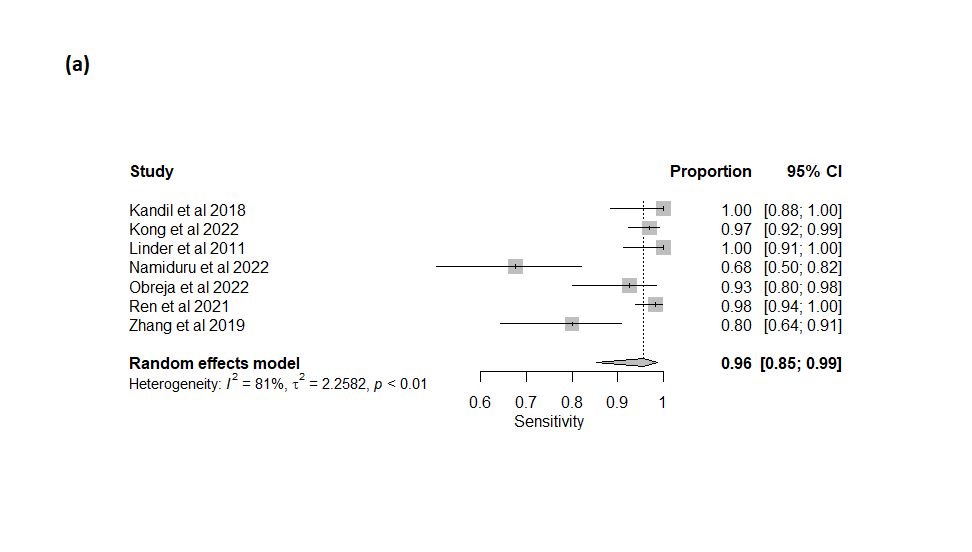

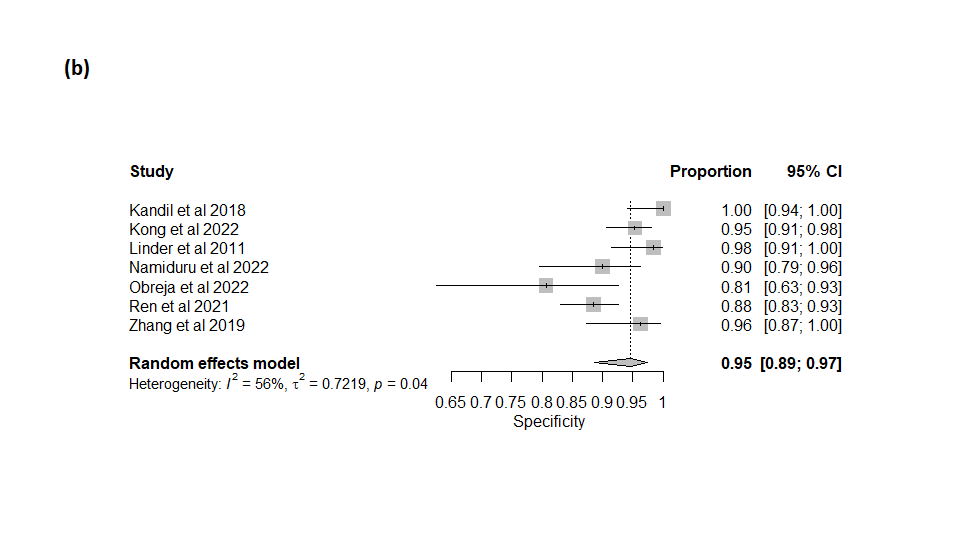


Figure S4. The Univariate Analysis for CSF HBP in Diagnosing CNS Infections Before Leave-one-out Test; (a) Forest Plot of Pooled Sensitivity; (b) Forest Plot of Pooled Specificity.


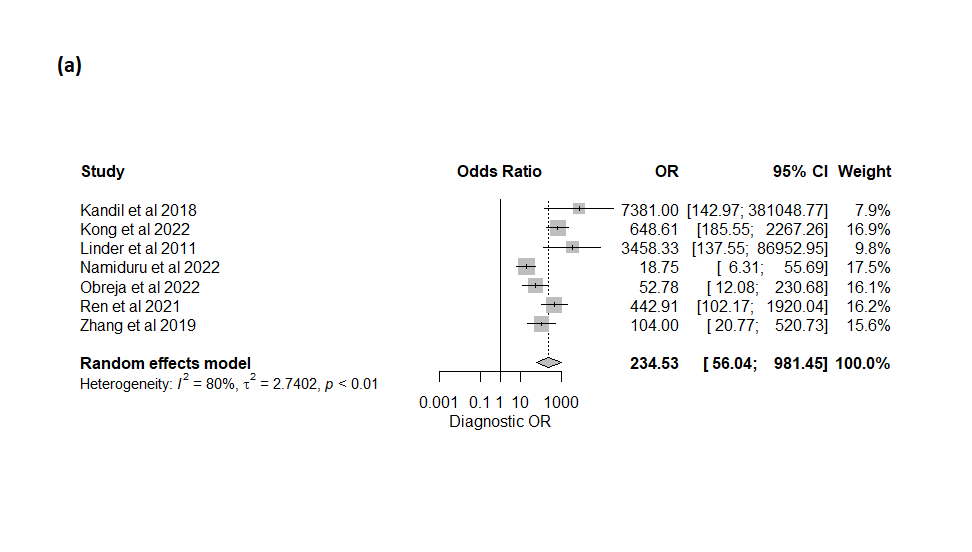

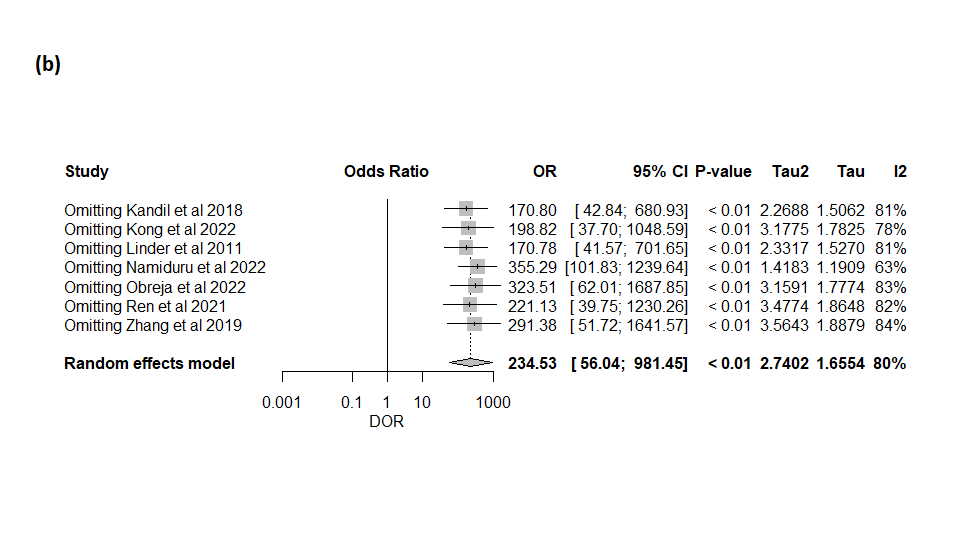


Figure S5. (a) Forest Plot of the diagnostic Odds Ratio (DOR) of CSF HBP for the Diagnosis of CNS Infections; (b) Forest Plot of the DOR of CSF HBP for the Diagnosis of CNS Infections After the Leave-one-out Test.

Table S2. Results of Meta-regression Analysis of Studies Investigating CSF HBP

| Covariate | I^2^ after (%) | Coefficient | SE of coefficient | p-value |
| --- | --- | --- | --- | --- |
| **Year** | 77.11 | -0.3490 | 0.2067 | 0.0913 |
| **Age** | 84.06 | -0.0070 | 0.0478 | 0.8830 |
| **Male** | 80.36 | 0.0107 | 0.0166 | 0.5193 |
| **Female** | 79.06 | 0.0102 | 0.0133 | 0.4409 |
| **HBP at baseline** | 67.21 | 0.0265 | 0.0120 | 0.0268 |
| **Cutoff** | 74.52 | 0.0640 | 0.0347 | 0.0647 |


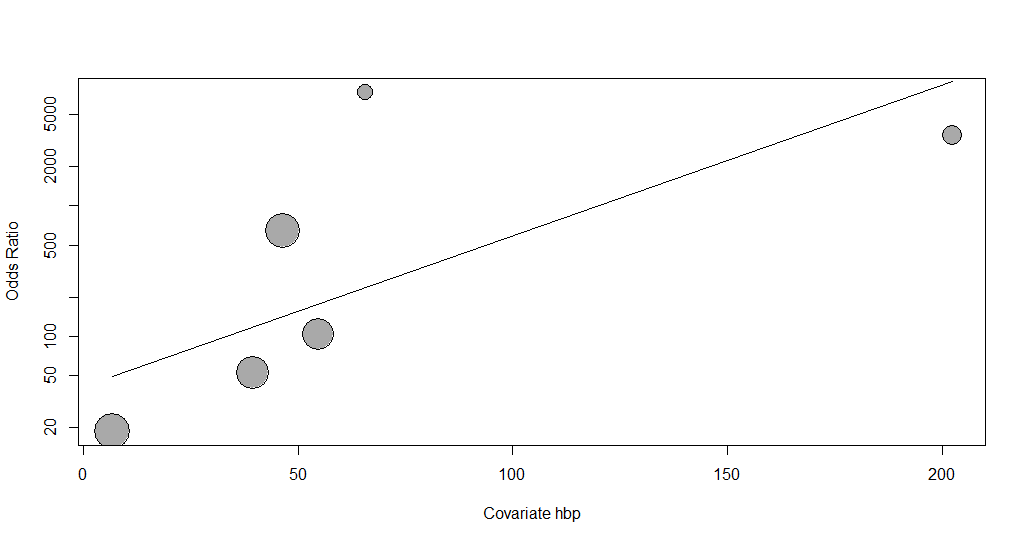


Figure S6. Deek's Funnel Plot Showing the Effect of HBP at Baseline on the Effect Size in Studies Investigating CSF HBP


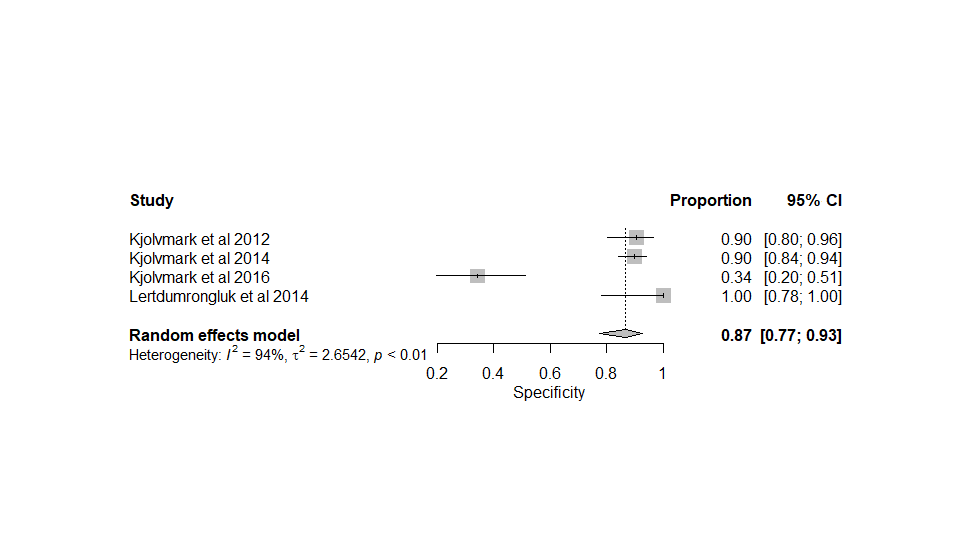


Figure S7. Forest Plot of Pooled Specificty of Urinary HBP in Diagnosing Urinary Tract Infections Before Leave-one-out Test.


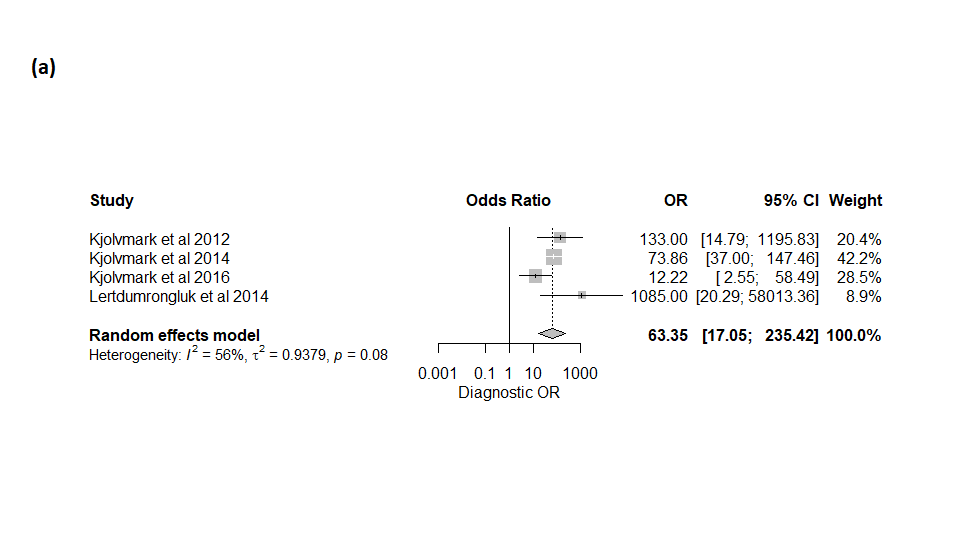

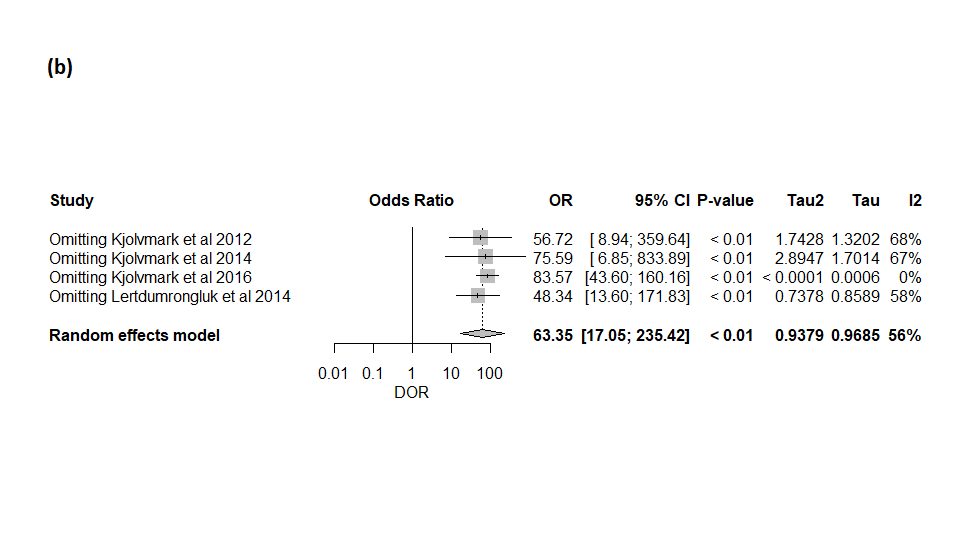


Figure S8. (a) Forest Plot of the diagnostic Odds Ratio (DOR) of Urinary HBP for the Diagnosis of Urinary Tract Infections; (b) Forest Plot of DOR of Urinary HBP for the Diagnosis of Urinary Tract Infections After the Leave-one-out Test.
